# Supplementary material for: Applicability of sentinel lymph node oriented treatment strategy for gallbladder cancer
Source: PLoS One. 2021 Feb 12;16(2):e0247079. doi: 10.1371/journal.pone.0247079 (PMC7880492; doi:10.1371/journal.pone.0247079)
Supplement: Attachment — Submitted filename: Manuscript edits_KS.docx [file pone.0247079.s001.docx]

Abstract

Line 39- change malignancy to malignancies

Line 52-53 reads as incomplete.

Line 87- would say examination of “the” SLN; and change malignancy to malignancies

Lines 90-92- major pathways to what? Assume means lymph drainage but would clarify

Line 96- Change according to accordingly

Line 104- change to patients who did not have data on location or had missing data on location

Line 129- would change to underwent laparotomy

Line 131- would add “were done” in T2h

Line 144- add status (“performance status”)

Line 153- surgical mortality “was” defined

Line 164- typically DFS is length of time from randomization (not applicable here) to recurrence of tumor OR death. Does not including patients who recurred OR died impact the study?

Line 174- were not significantly different

Line 178- would take out “the incidence of LN metastasis” as you already reported this above.

Line 184- what was the difference for these other post-op complications between T3p & T3h?

Line 186- remove each

Line 202- change “while” to “as compared to”

Line 214- on the peritoneal side

Lines 215- would change “especially among them” to “specifically” as this sets up for the breakdown of liver recurrence vs nodal

Line 216- change to “then that of”

Line 219- change to significant

Line 220- take out “the”

Line 234- change the extent to “extensive LND”

Line 238- did not mention

Line 240- take out “that”

Line 245- change “pf” to “of”

Line 254- take out “all”

Line 256- change to “few reports of a difference in”

Line 261- add “an” before independent poor

Line 262-263- change to something like “research into how GBC metastases through lymph nodes and the extent of LND are important”

Line 265- add conducted in or on; & remove “a” after relatively

Line 274- change to “how skip LN metastasis occurred”
